# Dedifferentiated Melanoma: A Diagnostic Histological Pitfall—Review of the Literature with Case Presentation

**DOI:** 10.3390/dermatopathology8040051

**Published:** 2021-10-15

**Authors:** Gerardo Cazzato, Lucia Lospalluti, Anna Colagrande, Antonietta Cimmino, Paolo Romita, Caterina Foti, Aurora Demarco, Francesca Arezzo, Vera Loizzi, Gennaro Cormio, Sara Sablone, Leonardo Resta, Roberta Rossi, Giuseppe Ingravallo

**Affiliations:** 1 Section of Pathology, Department of Emergency and Organ Transplantation (DETO), University of Bari “Aldo Moro”, 70124 Bari, Italy; anna.colagrande@gmail.com (A.C.); micasucci@inwind.it (A.C.); leonardo.resta@uniba.it (L.R.); roberta.rossi@policlinico.ba.it (R.R.); 2 Section of Dermatology, Department of Biomedical Sciences and Human Oncology (DIMO), University of Bari “Aldo Moro”, 70124 Bari, Italy; l.lospalluti@gmail.com (L.L.); paolo.romita@uniba.it (P.R.); caterina.foti@uniba.it (C.F.); aurorademarco94@gmail.com (A.D.); 3 Section of Ginecology and Obstetrics, Department of Biomedical Sciences and Human Oncology (DIMO), University of Bari “Aldo Moro”, 70124 Bari, Italy; francesca.arezzo@uniba.it (F.A.); vera.loizzi@uniba.it (V.L.); gennaro.cormio@uniba.it (G.C.); 4 Section of Legal Medicine, Department of Interdisciplinary Medicine, Bari Policlinico Hospital, University of Bari, 70124 Bari, Italy; sarasabloneml@gmail.com

**Keywords:** dedifferentiated melanoma, malignant melanoma (MM), immunohistochemistry, pitfall, diagnosis

## Abstract

Dedifferentiated melanoma is a particular form of malignant melanoma with a progressive worsening of the patient’s clinical outcome. It is well known that melanoma can assume different histo-morphological patterns, to which specific genetic signatures correspond, sometimes but not always. In this review we address the diagnostic difficulties in correctly recognizing this entity, discuss the major differential diagnoses of interest to the dermatopathologist, and conduct a review of the literature with particular attention and emphasis on the latest molecular discoveries regarding the dedifferentiation/undifferentiation mechanism and more advanced therapeutic approaches.

## 1. Introduction

Classically, melanoma has always been considered as “the great mime” for its intrinsic ability to disguise itself in different guises and imitate other types of neoplastic and non-neoplastic lesions [1]. This peculiarity constitutes the basic reason why the routine dermatopathologist must always resort to adequate immunohistochemical markers to exclude or confirm the diagnosis of malignant melanoma [1,2]. All this is not true in the case of a particular form of malignant melanoma, defined by various authors as “Dedifferentiated Melanoma” (DM) due to the characteristic of losing some or all of the melanocytic immunohistochemical markers [3]. The clinical and histopathological difficulty in recognizing and correctly diagnosing this entity has already been previously reported, although only in recent years has the advent of molecular biology and next generation sequences (NGS) contributed, in a fundamental way, to a better understanding of the dedifferentiated phenotype. In this paper, we present a case of dedifferentiated melanoma, we move between the bridle of differential diagnosis as we deal with “real life”, we conduct a review of the few cases reported in literature so far and, finally, we focus on the molecular characteristics of their own of this phenotype with particular attention to modern therapeutic treatments.

## 2. Materials and Methods

**Figure 1:**
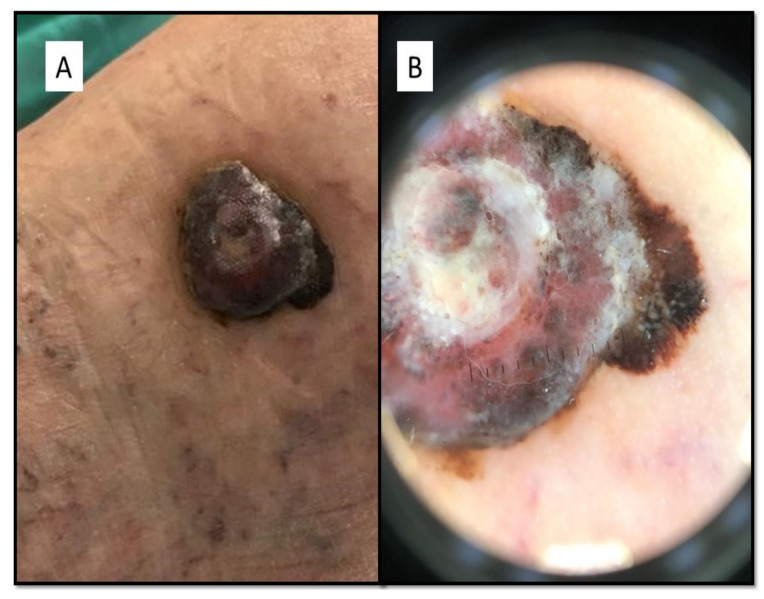
(**A**) Lesion consisted of a nodular part of about 3 cm in diameter and a flat, blackish, elongated part of about 1.5 cm. (**B**) Dermoscopically, the lesion was characterized by marked chromatic asymmetry, with radial striae and peripheral pigment escape, with irregular globules.

The patient we present was a 79-year-old woman, in fairly good general condition, who, in her medical history, reported the appearance of a flat pigmented lesion for many years, while she reported that the nodular part would have appeared around a year ago. From the clinical point of view, the lesion consisted of a nodular part of about 3 cm in diameter and a flat, blackish, elongated part of about 1.5 cm (Figure 1A). From the dermoscopic point of view, a marked chromatic asymmetry was noted: it progressed from a black-brown color to pink-white, with a whitish veil. The presence of radial striae, peripheral pigment escape with irregular globules was described (Figure 1B). The lattice was black in color, with irregular meshes, which abruptly interrupted itself in the periphery; furthermore, regression and vascular structures were present. By virtue of these characteristics, surgical removal with large resection margins was opted for and the sample was sent to the pathological anatomy laboratory. After adequate fixation in 10% buffered formalin, sampling, processing, and inclusion in paraffin, sections of about 6 microns thickness were prepared, stained with Hematoxylin–Eosin (H&E), and further sections kept for immunostaining with antibodies for melanocytic markers.

In addition to the presentation of this clinical case, we performed a review of the current literature using PubMed and Web of Sciences (WoS) as the database and as keywords “Dedifferentiated Melanoma” OR “Indifferentiated Melanoma” in combination with “Histology” OR “Histopathology” AND/OR “Dermoscopy”. This review was elaborated following the Preferred Reporting Items for Systematic Reviews and Meta-Analyses (PRISMA) guidelines [4]; eligible articles were assessed according to the Oxford Centre for Evidence-Based Medicine 2011 guidelines [5]. Review articles, meta-analyses, observational studies, case reports, survey snapshot studies, letters to the editor, and comments to the letters were all included. Other potentially relevant articles were identified by manually checking the references of the included literature. An independent extraction of articles was performed by two investigators according to the inclusion criteria. Disagreement was resolved by discussion between the two review authors.

## 3. Results

**Figure 2:**
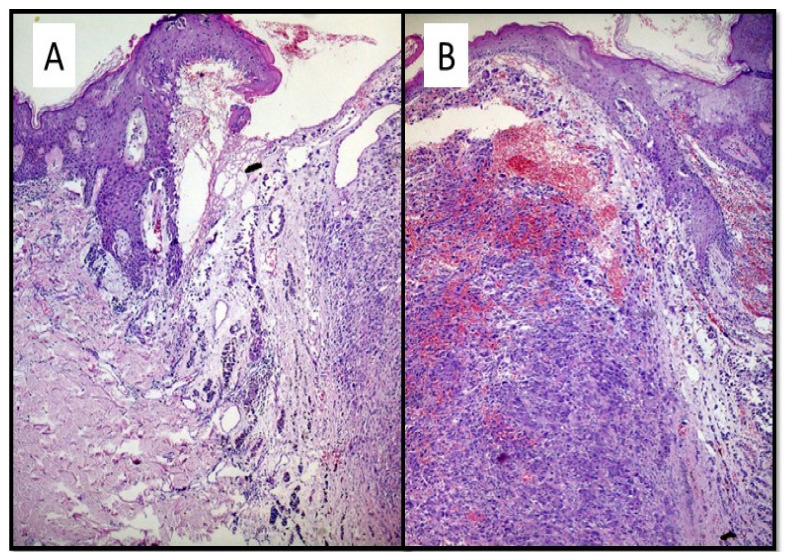

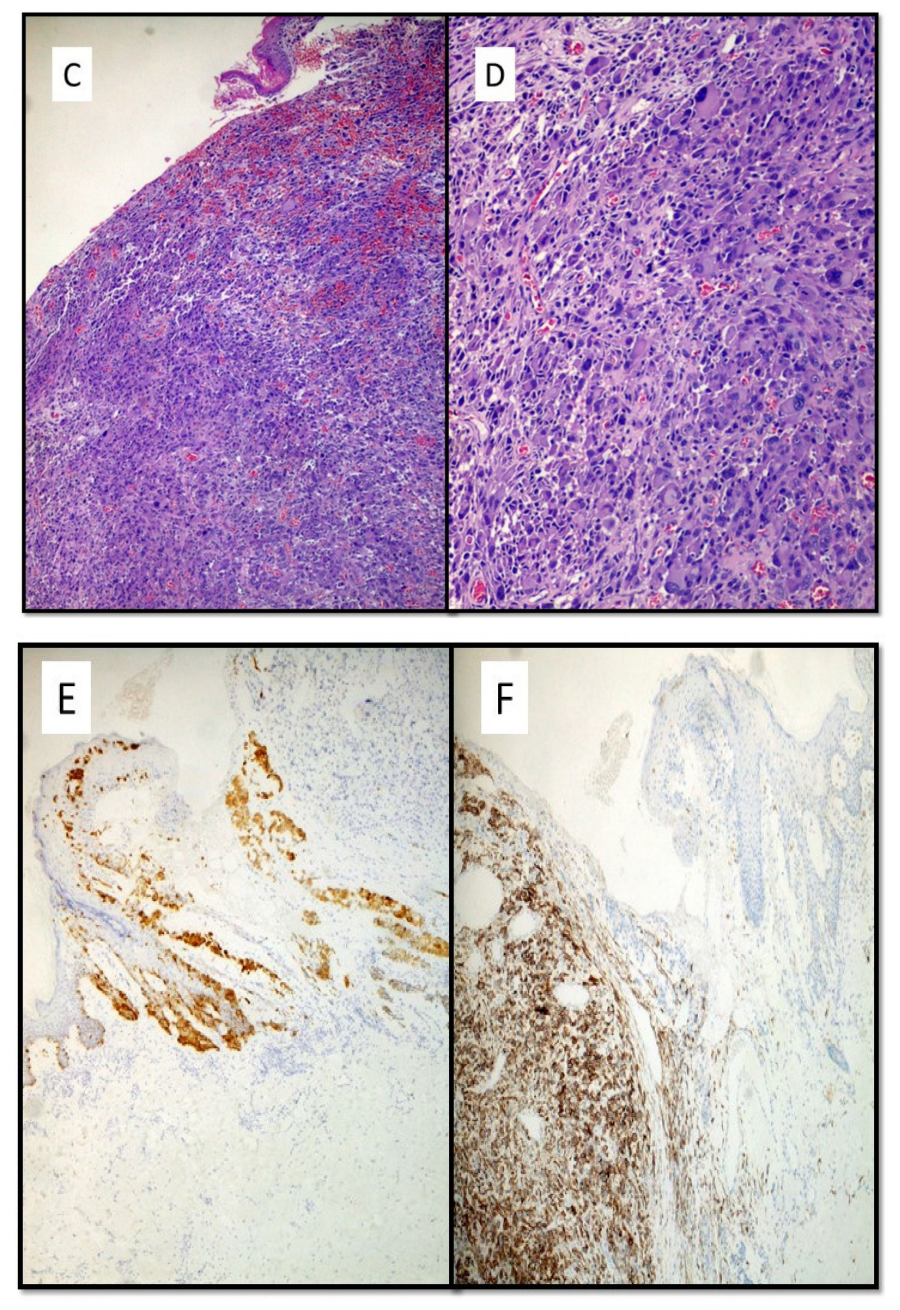
(**A**) The “shoulder” of the lesion was made up of atypical melanocytes, which ascended to the level of the dermoepidermal junction, morphologically suggestive of malignant melanoma. Note that the neoplastic cells had a tendency to invade the superficial/middle dermis (Hematoxylin-Eosin, Original Magnification: 10×). (**B**) Histological micrograph that highlights the two morphologically different components of dedifferentiated melanoma (Hematoxylin–Eosin, original magnification: 20×). (**C**,**D**) Nodule consisting of pleomorphic, atypical elements, with very numerous typical and atypical mitotic figures, eosinophilic intracytoplasmic paranuclear inclusions, nuclei with thinned chromatin and numerous central and peripheral nucleoli (Hematoxylin–Eosin, original magnification: 20× and 40×). (**E**) Immunostaining for HMB-45, which is strongly represented in the melanocyte proliferation constituting the “shoulder” of the lesion morphologically represented in (**A**). Note the total negativity of HMB-45 of atypical nodular proliferation (Immunohistochemistry, original magnification: 10×) (**F**) Immunostaining for CD10 strongly positive in the exophytic polypoid nodular component and negative in the shoulder of the lesion (Immunohistochemistry, original magnification: 10×).

On histological observation, two cell populations were described which constituted two (distinct?) formations: an exophytic polypoid nodule, consisting of pleomorphic, atypical elements, with very numerous typical and atypical mitotic figures, eosinophilic intracytoplasmic paranuclear inclusions, nuclei with thinned chromatin, and numerous central and peripheral nucleoli (Figure 2C). Furthermore, no pigment interspersed with this cell population was appreciated (Figure 2D). On the sides of the nodular lesion, elements frankly of a melanocytic nature were appreciated, with occasional pigment, which invaded the superficial and middle dermis (Figure 2A,B). The following findings were appreciated on immunohistochemical examination: the nodular component was almost entirely negative for S-100, Melan-A and HMB-45; only focally positive for SOX-10 (Figure 2E). In contrast, it was strongly positive for CD10 (Figure 2F). The “junctional” component, on the other hand, was strongly positive for S-100 protein, Melan-A and HMB-45, but negative for CD10 (Figure 2E).

The potential diagnoses were basically two: a “collision” lesion consisting of a melanoma and a malignant fibrohistiocytic neoplasm (such as for example the Atypical Fibroxanthoma), or a malignant melanoma that underwent almost total dedifferentiation, so as to almost entirely lose the common markers of melanocyte differentiation. A careful analysis and integration of morphology and immunohistochemistry allowed to describe the presence of focal clusters of SOX-10 and S-100 protein positive melanocytes within the nodular lesion proper (area of transition). For this reason, this lesion was diagnosed as entirely melanoma with a dedifferentiated component, strongly expressing CD10.

Furthermore, for definitive confirmation, we performed Next Generation Sequencing analyses at an external center that revealed BRAFV600K mutation, supporting the diagnosis of dedifferentiated melanoma.

By virtue of the rarity and the diagnostic challenges that DM poses, we have conducted a careful review of the current literature, in order to improve the characterization, understanding, and knowledge of this potential diagnostic pitfall.

**Figure 3:**
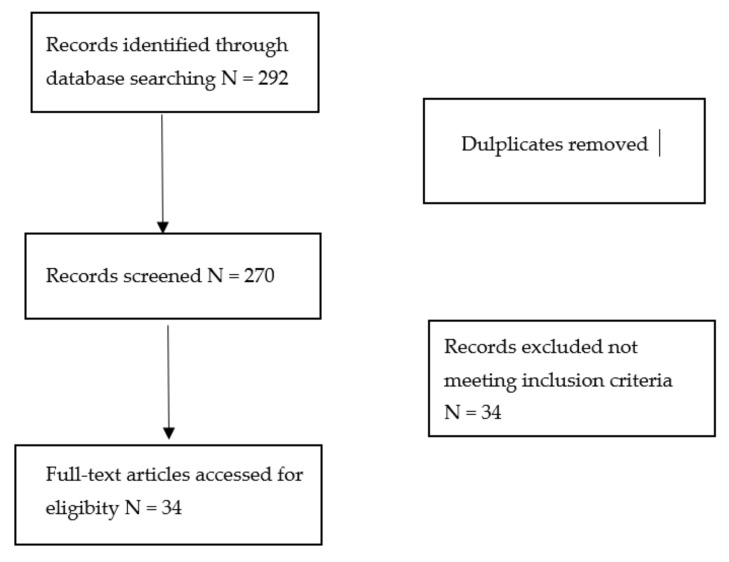
Literature search and article selection according to PRISMA guidelines.

The research of the literature made it possible to highlight 292 scientific articles using the keywords mentioned above. Of these, duplicate ones (*n* = 22) and those whose inclusion criteria were not known (*n* = 34) were excluded. In addition, articles that did not primarily examine the topic of “dedifferentiation” were eliminated. In the end, therefore, 34 scientific articles were included (Figure 3).

## 4. Discussion

A malignant melanoma may be able to simulate various and different neoplasms: the potential of being misunderstood with other malignancies is well known [1,2]. In this corollary, dedifferentiated melanoma turns out to be a very aggressive form, with little tendency to medical response [3]. Although rare, the dedifferentiation mechanism has been studied in different sets of neoplastic pathologies, and it is recognized quite clearly that in addition to creating difficulties in the correct histopathological diagnosis of the entity, DM poses problems of therapeutic response both to traditional therapy and to immunotherapy (so-called cross-resistance) [6]. Although the histological diagnosis of malignant melanoma is known to be difficult, this is even more true in the case of DM, as the morphological characteristics on the one hand and the loss of one or all of the markers of melanocytic differentiation (such as Melan-A, HMB-45, SOX-10 and MITF), poses major diagnostic challenges for, among others, high-grade sarcomas or carcinomas [7,8]. Additionally, in our case, the dedifferentiation within the morphologically characterizable malignant melanoma created some diagnostic questions regarding the possibility that it was a collision lesion rather than a melanoma with a real portion of dedifferentiation. This issue has been extensively discussed in the literature [7,8,9,10], and a case has been reported very recently by *Saldana* et al. [11] in which the amplification in FISH of the MDM2 gene was described for the first time in a lesion of a 73-year-old subject, which had led, in the first instance, to hypothesize a liposarcoma. A careful analysis involving also the determination of BRAFV600 allowed to reach the correct diagnosis of DM.

In our case, only a careful evaluation of focal positivity of a cluster of cells for SOX-10 allowed us to diagnose DM and not of other entities, but the simultaneous expression of immunohistochemical markers such as CD10 posed greater difficulties in the diagnosis.

In the last 10 years, various authors have tried to shed light on what may be the potential biological pathways that melanocyte cells follow until they lose the common immunohistochemical markers [6,7,8,9,10,11,12,13,14,15,16,17,18,19,20,21,22,23,24,25,26,27,28,29,30,31,32,33,34]. Therefore, the concept of “phenotypic plasticity” of melanocyte cells has been developed, as it has been demonstrated that the microenvironment where melanocytes operate is able to bi-directionally influence the following phenotype: in particular, the study of melanocytes has been deepened, inducing the transcription factor (MITF) whose expression (also detectable in immunohistochemistry) was correlated with a different biological behavior of melanoma cells. Depending on a greater or lesser expression of MITF, the clones of melanoma have recently been differentiated into: highly proliferative/minimally invasive and low proliferative/highly invasive [6,11]. In addition to MITF, other melanocytic genes (such as TYR, DCT, MART-1) are also upregulated in the proliferative phenotype of melanomas. Conversely, in the invasive genetic signature MITF and other genes (such as INHBA, COL5A1 and SDERPINE1) are involved in modifying the extracellular environment [6,12,13,14,15,16,17,18,19,20].

Furthermore, various authors have shown how the dedifferentiation mechanism is a predictor of poor response to target therapy: for example, although the discovery that the BRAF mutation may be the occasion for molecular targeted therapy, patients with DM appear to have little clinical benefit, both in terms of PFS and OS [21,22,23,24].

From a strictly dermatopathological point of view, a careful evaluation of cell morphology and an inconclusive immunohistochemistry for a specific entity are the starting points from which to start: various authors [25,26,27,28], for example, consider it important, when it is not possible to be sure only with routine diagnostic techniques, carry out molecular investigations for the BRAF mutation, so as to be sure of being faced with a case of DM [29,30,31]. Alkhasawneh et al., in 2019, reported the case of a 52-year-old woman, previously operated on for breast cancer and previous melanoma resulting in pT1b, who presented with a chest lesion that had entirely lost all melanocytic immunohistochemical markers, and expressed (aberrantly) only GATA-3. The diagnosis of DM was made only after careful analysis of the mutation for BRAFV600K [32].

Finally, it is important to underline how the discovery and deepening of the mechanisms of regulation of melanogenesis in mammals [33,34] have clarified that melanocytes are endowed with both a responsive function towards signal molecules (paracrine regulation) and autocrine regulation. This field is very interesting in trying to understand how in malignant melanomas these “physiological” pathways are dysregulated, and we receive lesions with large and abundant extruded melanic pigment.

## 5. Conclusions

Dedifferentiated melanoma is a unique clinical/biological entity, which continues to pose significant diagnostic challenges. It is quite understood that there are difficulties in differential diagnostics with other malignant neoplastic lesions such as undifferentiated carcinomas and sarcomas; dedifferentiation is only a single epiphenomenon of an underlying biological heterogeneity that governs the behavior and clinical aggression of the disease; however, dedifferentiation is a marker of cross-resistance to target therapy and immunotherapy.

## Data Availability

Not applicable.
